# Distribution and diversity of eukaryotic microalgae in Kuwait waters assessed using 18S rRNA gene sequencing

**DOI:** 10.1371/journal.pone.0250645

**Published:** 2021-04-26

**Authors:** Vinod Kumar, Sabah Al Momin, Vanitha V. Kumar, Jasim Ahmed, Lamya Al-Musallam, Anisha B. Shajan, Hamed Al-Aqeel, Hamad Al-Mansour, Walid M. Al-Zakri

**Affiliations:** Environment and Life Sciences Research Center, Kuwait Institute for Scientific Research, Kuwait, Kuwait; Bharathidasan University, INDIA

## Abstract

The microbial communities play a crucial role in ecosystem functioning through interactions among individuals and taxonomic groups in a highly dynamic marine ecosystem. The structure and functioning of the microbial communities are often influenced by the changes in the surrounding environment. Monitoring the microbial diversity of the marine ecosystem helps to understand spatial patterns of microbial community and changes due to season, climate, and various drivers of biological diversity. Kuwait is characterized by an arid environment with a high degree of temperature variation during summer and winter. Our understanding of spatial distribution patterns of microbial communities, their diversity, and the influence of human activities on the degree of changes in the diversity of the microbial community in Kuwait territorial waters remain unclear. In this study, we employed 18S rRNA sequencing to explore marine microalgal community composition and dynamics in seawater samples collected from Kuwait waters over two seasonal cycles across six locations. A total of 448,184 sequences across 36 replicates corresponding to 12 samples from six stations were obtained. The quality-filtered sequences were clustered into 1,293 representative sequences, which were then classified into different eukaryotic taxa. This study reveals that the phytoplankton community in Kuwait waters is diverse and shows significant variations among different taxa during summer and winter. Dinoflagellates and diatoms were the most abundant season-dependent microalgae taxa in Kuwait waters. *Alexandrium* and *Pyrophacus* were abundant in summer, whereas *Gonyaulax* was abundant during the winter. The abundance of *Coscinodiscus* and *Navicula*, of the diatom genera, were also dependent upon both seasonal and possible anthropogenic factors. Our results demonstrate the effectiveness of a sequencing-based approach, which could be used to improve the accuracy of quantitative eukaryotic microbial community profiles.

## Introduction

Billions of marine microbes are present in a liter of seawater; about 70% of the oceanic biomass is derived from marine microorganisms [1]. These microorganisms, including protists, bacteria, fungi, and viruses, fundamentally influence the ocean’s ability to sustain life on earth, playing a crucial role in the recycling of nutrients in the ecosystem. The microflora of oceans make up a vast biological network, and their behaviour influences how the oceans respond to broader environmental changes [2]. Thus these microorganisms are of paramount importance in global ocean change [3].

Marine metagenomics-based bioprospecting has gained serious attention because of the possibility of identifying various sources of enzymes that are relatively more stable and useful than the corresponding plant- or animal-derived enzymes [4,5]. Several marine microbiome-derived bioactive compounds have been explored for their potential biotechnological and pharmaceutical applications [6,7]. The largest primary biomass, the microalgae, is of particular interest due to its applications in various biotechnological fields [8,9]. Furthermore, marine cyanobacteria also serve as a resource for several useful enzymes and genes [10].

The availability of metagenomes will eventually help in the growth and development of marine biotechnology. The advanced technologies in current genomics can be applied to explore microbial communities in ocean ecology [11]. Several genomic studies have focused on ocean photosynthetic microbes [12]. Louca et al. (2016), analyzed the taxonomic and functional community profiles across the global ocean [13] and, due to the increasing demand for microalgal applications in the production of marine natural products, several omics datasets have also been generated [8].

The marine microbiome is also of great interest in the food and bioprocessing industry [14–16]. Microalgae production has the potential to enhance the sustainability of global fisheries and aquaculture [17]. Aquaculture is a fast-growing industry in the state of Kuwait, with a total production of 197 metric tons in the year 2018 [18], which only accounts for about 20% of the local consumption, indicating a huge potential to scale up. Several microalgae species, e.g., *Nannochloropsis*, *Chlorella*, *Chaetoceros*, *Tetraselmis*, and *Isochrysis* are currently being used to feed rotifers, which constitute the main live feed for rearing larvae in aquaculture production [19–21]. The marine microbiome studies in Kuwait waters could help in the management of marine bioresources, identification of climate-adapted microalgae species for use in food, feed and energy sectors.

The marine environment of Kuwait bay is often affected by various anthropogenic activities. The petrochemical industry, power plants, desalination plants, and sewage outlets near urban settlements often cause a negative impact on the marine environment. It has been reported that over 53,000 oil tankers move every year in the Arabian Gulf, transporting crude oil [22], and, their untreated ballast water has a negative effect on the marine species and aquatic environment [22], causing an increase of non-native organisms, disrupting the food chain, and causing fatal diseases. The gulf countries heavily rely on the desalination of seawater to meet freshwater demands. The desalination plants in the Gulf Cooperation Council (GCC), discharge around five million cubic meters of brine water per day, approximately half the total global brine discharge, into the waters of the Arabian Gulf [23].

Despite its vast diversity, the marine microflora in Kuwait is poorly understood. The marine environment of Kuwait is markedly affected by industrialization and urbanization [24]. Kuwait Bay is periodically monitored at designated stations by systematic sampling and continuous research by various research groups.

The selected stations in this study for microbial community analysis represent the greater part of Kuwait bay from north to south, with varying water depths ranging from 5m to 29m. The temperature and salinity of the seawater varied from 17.5 to 30°C and 38 to 44 parts per thousand (ppt), respectively [23,25,26]. The water quality of Kuwait bay is often affected by effluents from sewage, power plants, desalination units, pollutants like oil, petroleum hydrocarbons, trace metals, suspended particles, and nutrients. These anthropogenic activities have a profound effect on the water quality of Kuwait bay, especially the sampling stations located in the close vicinity of the coast and near the urban settlement. It is evident from the previous reports that the station C and K6 are more prone to domestic and industrial effluents and the main centre of recreation. Both these locations receive municipal waste from the densely populated Kuwait City [25,27] in addition to effluents from various industrial units, power and desalination plants, and vessels entering Kuwait Port [25]. Stations A and B are located in the north of Kuwait in a region that receives runoff from the Shatt Al-Arab river. The water discharge from the Shatt Al Arab river is a key point source of a nutrient influx in the northern part of Kuwait Bay. This generates a gradient of nutrient level from north to south in Kuwait bay. The salinity in both of these stations is relatively low during spring and early summer [23]. Stations A and B are subjected to moderate levels of anthropogenic changes. Stations 3 and 18 are located away from the coast, in the least polluted offshore, with minimal anthropogenic effect.

With the development of next-generation high-throughput sequencing technologies, it could be possible to catalogue the microbial niche and explore its diversity across ocean environments. Further, the technology is increasingly used to understand microbial communities, ecosystem functioning and biodiversity. The 18S rRNA genomic region has widely been used to explore the eukaryotic marine microbiome [28]; for example, the Ocean Sampling Day (OSD) project examined the microalgae in several marine locations [29].

The current study is a metagenomic approach to assess the eukaryotic microalgal distribution and their diversity at different stations in Kuwait waters across summer and winter. The study provides the first insight into the marine eukaryotic microbiome in this region.

## Materials and methods

### Sample collection and DNA isolation

Seawater samples were collected from six different stations (K3, K18, KA, KB, K6, and KC) in Kuwait waters and are detailed in **Table 1.** From each sampling site, a 500-ml bulk water sample was collected in plastic bottles by oblique towing using a 20-μm phytoplankton net. The plankton were funnelled into a collection bottle, from which the concentrated population was bulked to obtain three replicates. The sampling was performed at each location during the morning hours. The collected samples were transported to the laboratory in an icebox to maintain the cold temperature.

**Table 1:** Details of the samples and collection stations.

Station Name*	Alternative Name of the Station^#^	Latitude and longitude	Season of sample collection
KS3	3	29°25’00.0"N 48°30’00.0"E	Summer
KS18	18	29°03’00.4"N 48°30’00.2"E	Summer
KW3	3	29°25’00.0"N 48°30’00.0"E	Winter
KW18	18	29°03’00.4"N 48°30’00.2"E	Winter
KSA	A	29°36’00.0"N 48°10’00.0"E	Summer
KSB	B	29°31’00.1"N 48°15’00.0"E	Summer
KWA	A	29°36’00.0"N 48°10’00.0"E	Winter
KWB	B	29°31’00.1"N 48°15’00.0"E	Winter
KS6	K6	29°27’00.0"N 47°58’00.0"E	Summer
KSC	C	29°25’00.4"N 47°50’00.6"E	Summer
KW6	K6	29°27’00.0"N 47°58’00.0"E	Winter
KWC	C	29°25’00.4"N 47°50’00.6"E	Winter

*K: Kuwait, W: Winter, S: Summer

^#^station name used in the published literature [23,25,30,31].

Total DNA isolation was performed on seawater samples on the day of collection. The water sample was centrifuged at 10,000 g for 20 minutes using the high-speed centrifuge (Gyrozen 2236R, Gyeonggi-do, Republic of Korea). The pellet was suspended in 5 ml of distilled water and centrifuged further at 10,000 g for 15 minutes. The supernatant was discarded, and the pellet was used for DNA extraction. Total genomic DNA was isolated using a Power Soil DNA Isolation Kit (MO BIO Laboratories, Carlsbad, CA) following the user manual. The concentration and purity of the eluted DNA samples were measured on a Nanodrop1000 UV/VIS Spectrophotometer (Thermo Fischer Scientific Waltham, MA, USA). The integrity of the DNA was confirmed by agarose gel electrophoresis using 0.8% agarose gel.

### Sequencing of the 18S rRNA gene

The 18S rRNA gene region was amplified with forward (GCGGTAATTCCAGCTCCAA) and reverse (AATCCRAGAATTTCACCTCT) primer sequences using the HotStarTaq Plus Master Mix Kit (Qiagen, USA) under the specified conditions: 94°C for 3 minutes, followed by 28 cycles of 94°C for 30 seconds, 53°C for 40 seconds and 72°C for 1 minute, after which a final elongation step at 72°C for 5 minutes was performed. After amplification, PCR products were checked on a 2% agarose gel to determine the success of amplification and the relative intensity of bands. The samples were purified using the calibrated Ampure XP beads and the purified PCR product was used to prepare the DNA library by following the Illumina TruSeq DNA library preparation protocol. The sequencing was performed at the Beijing Genomics Institute (BGI), Hong Kong, on a HiSeq platform following the manufacturer’s guidelines. Each sample was sequenced as a paired-end set of reads with a read length of 200–250 bp, and the data was obtained in fastq files. The raw data has been submitted to NCBI-SRA (PRJNA633289).

### Analysis of 18S rRNA gene sequencing data

The raw 18s rRNA data were checked for quality using FastQC v0.10.1 [32]. *DADA2* pipeline implemented in QIIME2 [33] was used for detecting and correcting Illumina amplicon sequence data. This quality control process filters any phiX reads (commonly present in marker gene Illumina sequence data) that are identified in the sequencing data, and chimeric sequences, resulting in filtered non-chimeric sequences. No truncation was done for the forward reads as the sequence quality was good, whereas reverse reads were truncated at the 235^th^ base to filter the bad quality bases. The filtered reads were joined and clustered to form OTUs or sequence variants with 100% similarity.

The q2-feature-classifier plugin implemented in QIIME2 was employed to train the classifier and classify the representative sequences. The 18s ribosomal RNA gene sequences corresponding to eukaryotic species were downloaded from the SILVA database [34]. Thereafter, the primer sequences were used for extracting the sequence regions with a minimum length of 100 and a maximum length of 500 from the complete database sequences. The extracted sequences and the corresponding SILVA taxonomy were used to train the Naïve Bayes classifier. The representative sequences were then classified, based on the trained classifier, into different eukaryotic species.

Network analysis among the dinoflagellate genera was performed using the CoNet v1.1.1 [35] application in the Cytoscape v3.7.0 [36]. Count matrices with the count for each genus in each sample were used followed by ‘*col_norm*’ and ‘*row_mincc*:*4*’. Spearman’s rank correlation coefficient of 0.3 was used and Fisher’s Z test *P* value threshold of 0.05 was considered to be statistically significant.

### Statistical analysis

The representative sequences were aligned using the *q2-phylogeny* plugin in QIIME2. The Mafft algorithm [37] was used to perform the multiple sequence alignment of the representative sequences. The aligned sequences were filtered for the highly variable regions, followed by the construction of a phylogenetic tree using the FastTree program [38]. The plugin *q2-diversity* was used for diversity analysis, including alpha and beta diversity. Alpha diversity indices, such as Shannon, Faith’s phylogenetic diversity, Pielou’s evenness, and beta diversity indices, such as UniFrac distance, were calculated using the rooted phylogenetic tree and a sampling depth of 7,700, corresponding to the lowest number of sequences in any sample. The differential abundance of the eukaryotic taxa was performed using the edgeR tool [39], and a *P*-value of <0.05 was considered statistically significant.

## Results

### Sequencing and filtering of reads

The sequencing of seawater samples obtained from Kuwait waters (**Table 1**) resulted in 448,184 sequences across 36 replicates corresponding to 12 samples (triplicates per sample were sequenced) from six stations. After multiple filtering steps, including denoising, quality score filtering, and chimera removal, approximately ~77% of the good quality data (343,439 sequences) remained. Most of the sequences were removed during the chimera filtering step and merging of paired-end reads (**Table 2**). On average, 28,620 sequences were obtained per sample. After filtering, the highest number of raw sequences retained for the KW6 sample (88.3%), whereas KS3 had the lowest number of sequences (64.2%). The number of raw and filtered reads per replicate is provided in **S1 Table**. The rarefaction analysis indicated the sequencing depth to be sufficient to cover the eukaryotic microbial diversity of all samples (**S1 Fig**). The quality-filtered sequences were clustered into 1,293 sequence variants/representative sequences, which were then classified into different eukaryotic taxa.

**Table 2:** Summary of raw and filtered reads.

Sample name	Raw read	Filtered reads	Denoised read	Merged sequence	Non chimeric sequence	% of Non-chimeric sequence
KS3	37,486	36,662	36,092	31,589	24,054	64.2
KS18	37,130	36,239	35,963	34,490	30,297	81.6
KW3	37,273	36,298	35,811	33,761	30,674	82.3
KW18	37,486	36,524	36,058	34,013	30,371	81.0
KSA	37,178	36,183	35,225	31,547	25,099	67.5
KSB	37,126	36,164	35,707	32,529	27,343	73.6
KWA	37,593	36,660	35,764	33,499	30,804	81.9
KWB	37,395	36,365	35,599	32,325	28,079	75.1
KS6	37,554	36,489	35,954	32,708	26,023	69.3
KSC	37,001	36,196	35,908	34,463	30,879	83.5
KW6	37,592	36,558	36,191	34,629	33,192	88.3
KWC	37,370	36,748	36,344	34,008	26,624	71.2
**Total**	**448,184**	**437,086**	**430,616**	**399,561**	**343,439**	**76.6**

### Alpha and beta diversity analysis

**Fig 1:**
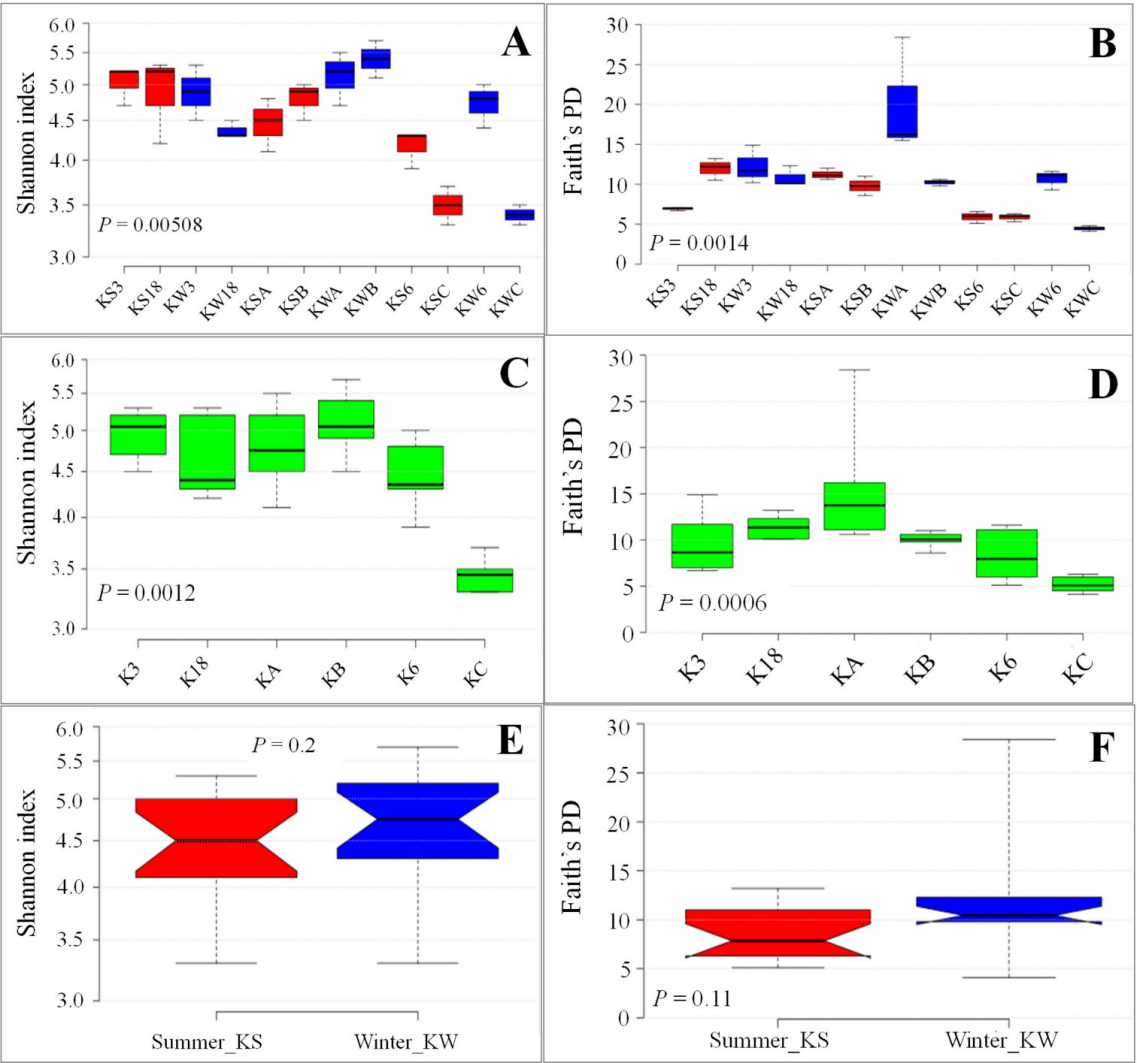
Alpha diversity indices across sample groups. A, C, and E: Shannon diversity indices across samples, stations, and seasons, respectively. B, D, and F: Faith’s phylogenetic diversity indices across samples, stations, and seasons, respectively.

The Shannon Index and Faith’s PD indicated that the water samples collected from the stations during summer and winter were significantly different in their diversity. Furthermore, the samples collected from Kuwait Bay with moderate (KA and KB) or low levels of anthropogenic activity (K3 and K18) were different from the samples from areas of relatively high anthropogenic activity (K6 and KC), as illustrated in **Fig 1**. Additionally, the seasonal difference (summer vs. winter) was obvious in eukaryotic diversity. The Shannon diversities of the samples collected from stations K3 and K18 during winter were higher than those of samples collected from the same stations during summer, although the differences were not statistically significant (**S3 Table**). However, there was a reverse trend for Shannon diversity for the samples collected from the stations KA and KB, where the diversity was higher during the winter (**Fig 1A**). Faith’s phylogenic diversity (PD) followed a similar trend with a few exceptions. For example, Faith’s PD for one of the samples collected from Station C was lower than that of all the samples collected from the same station during summer or winter (**Fig 1B**). Furthermore, the samples collected from stations K6 and KC exhibited different values during different seasons. The samples collected from station K6 during winter (KW6) had higher diversity, whereas the samples collected from station KC showed higher diversity during the summer. Details of the different diversity indices for individual replicates are provided in **S2 Table.** The statistical significance of the diversity indices between the samples collected from the same stations during the summer and winter seasons is provided in **S3 Table**. In addition, we observed marginally higher diversity in the samples collected during the winter than those collected during the summer, irrespective of the station type, although the difference was statistically insignificant (**Fig 1E and 1F**).

**Fig 2:**
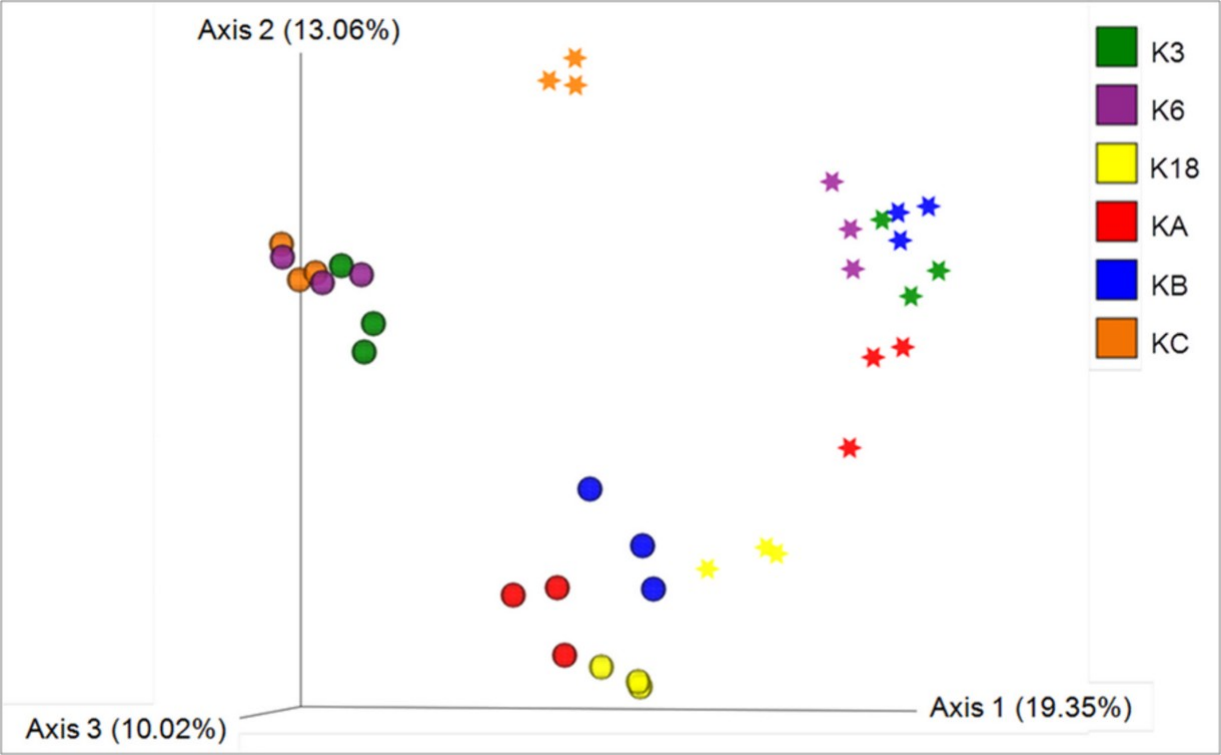
Principal component analysis of samples based on unweighted UniFrac distance matrix. Different colors indicate samples from different stations. **Sphere**: Samples collected during the summer season; **Star**: Samples collected during the winter season.

The clustering of samples based on an unweighted UniFrac distance matrix showed distinct clustering based on the season (**Fig 2**), indicating a differential eukaryotic microbial abundance during summer and winter. Furthermore, the samples collected during summer clustered together, based on the degree of anthropogenic activity, except for those from Station K3. For example, the samples collected from the stations that were more affected by anthropogenic activity (K6 and KC) clustered separately from the samples collected from stations with moderate or no anthropogenic effects. The samples collected from Station K3 during summer, however, clustered with the samples that were obtained from the stations with high anthropogenic changes during the same season. Also, the samples collected from Station K18 during the winter showed a remarkable resemblance to the samples collected from a variety of stations (KB, KA, K18) during the summer.

### Eukaryotic communities at different stations in Kuwait Bay

**Fig 3:**
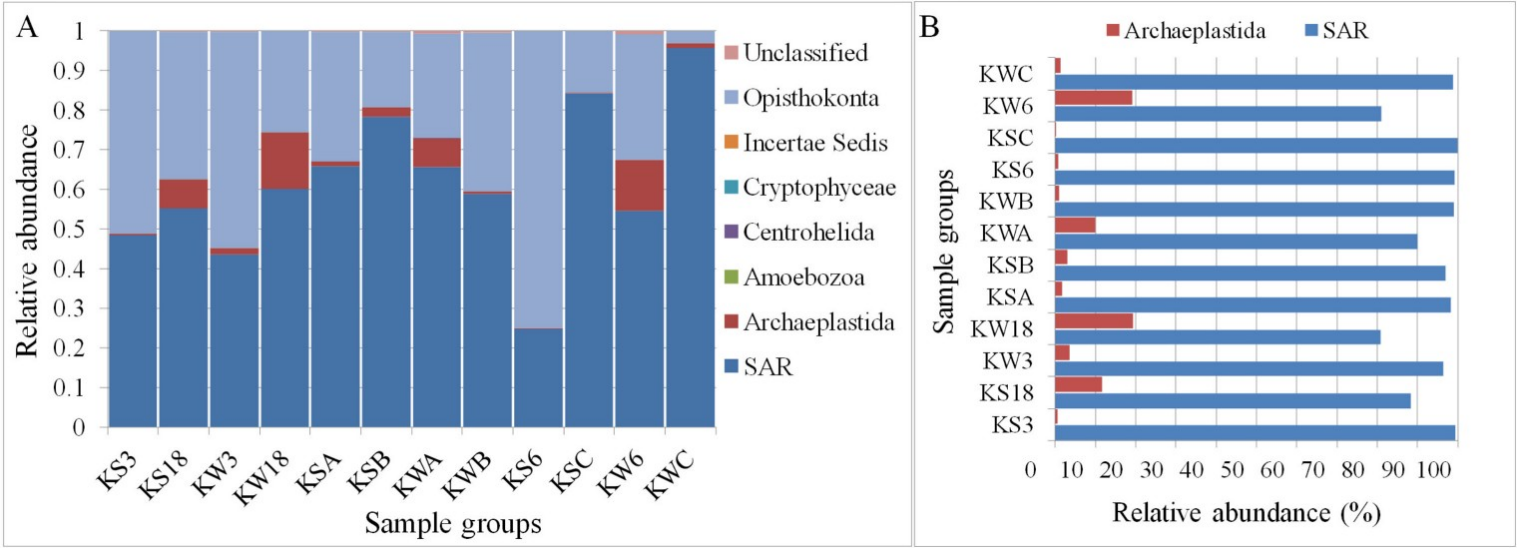
The relative abundance of all eukaryotic groups (A) and algal groups (B) at different stations in Kuwait waters.

Of the 343,439 sequences, a total of 342,614 were classified into seven different eukaryotic groups (**Fig 3A**). Among these, the SAR group (Stramenopiles, Alveolates, and Rhizaria) had the highest abundance with 61.4% (210,848 sequences), followed by Opisthokonta with 34% (116,600 sequences). Opisthokonta is a large super-group of eukaryotes, which includes animals, fungi, flagellates, amoeboids, and protists. Among the other groups, Amoebozoa, Centrohelida, and Cryptophyceae, each had fewer than ten sequences and was present in a maximum of two stations. Hence, they were considered artefacts. In addition, four sequences were classified as Incertae Sedis, which includes organisms that cannot be assigned to any group. Thus, Archaeplastida and SAR groups, covering 66% of the total sequences (225,989 sequences), were examined further to characterize the microalgae-related taxa at different sampling stations in Kuwait Bay (**Fig 3B and S1 File**). The Archaeplastida group comprises autotrophic eukaryotes, including red and green algae, unicellular algae (Glaucophytes), and land plants. The SAR super-group includes Stramenopiles (heterokonts), Alveolates, and Rhizaria. These subgroups include different algal and fungal taxa.

### Abundances of algal taxa

**Fig 4:**
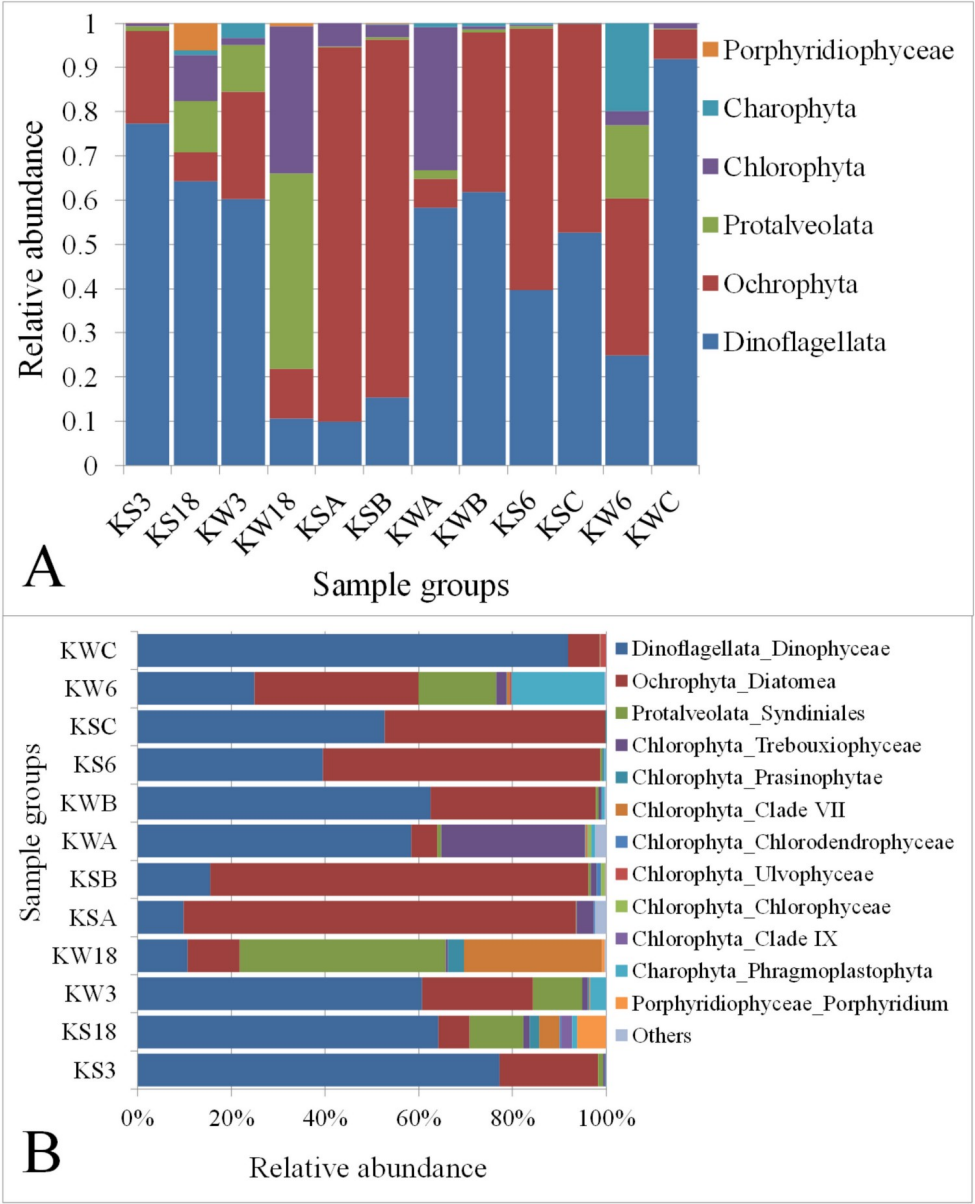
Relative abundance of (A) eukaryotic and (B) algal taxa across the sampling stations. KS: Samples collected during summer; KW: Samples collected during winter. ‘Others’ include taxa with <0.1% abundance.

The SAR group was classified into three algal phyla/subphyla (Dinoflagellata, Ochrophyta, and Protalveolata). Similarly, Archaeplastida was classified into Chlorophyta, Charophyta, and Porphyridiophyceae. Among the algal taxa, Dinoflagellata was the most abundant (50%), followed by Ochrophyta (34%) (**Fig 4A** and **S2 File)**. The sampling stations showed a high degree of variation in the abundance of these taxa. For example, station C during winter had the highest abundance of Dinoflagellata. However, the dinoflagellate abundance at Stations KW18, KSA, and KSB was comparatively lower. Ochrophyta was the most abundant algal taxon in Stations KSA, KSB, and KS6 during the summer season (84.8%, 80.8%, and 59.2%, respectively). Interestingly, in Station KW18, Protalveolata was the most abundant taxon (44.2%), followed by Chlorophyta (33.4%). The phylum Charophyta was very abundant in the samples collected from Station K6 (19.8%) during winter. However, in other stations, it was either undetectable or found at very low levels.

Although the overall abundance of the phylum Chlorophyta, among the six classified algal groups, was about 6% (**Fig 4A**), it was found to be the most diverse phylum with eight classes/sub-groups (**Fig 4B**). However, the organisms belonging to the class Dinophyceae had the highest abundance in most samples. The algae class Diatomea was found to be more abundant in the summer samples collected from the Stations KSA, KSB, KS6 and KSC compared with the winter samples collected from those stations, as well as from other stations. The organisms from the algal class Syndiniales were found to be relatively more abundant in four sample groups (KS18, KW3, KW8, and KW6) with an average abundance of 20%, whereas in all the other stations, their abundance was less than 1%. The subdivision Phragmoplastophyta was found to be more abundant (20%) in the samples collected from Station K6 in winter (KW6) compared with other sample groups.

### Seasonal (summer vs. winter) variation in the algae community

**Fig 5:**
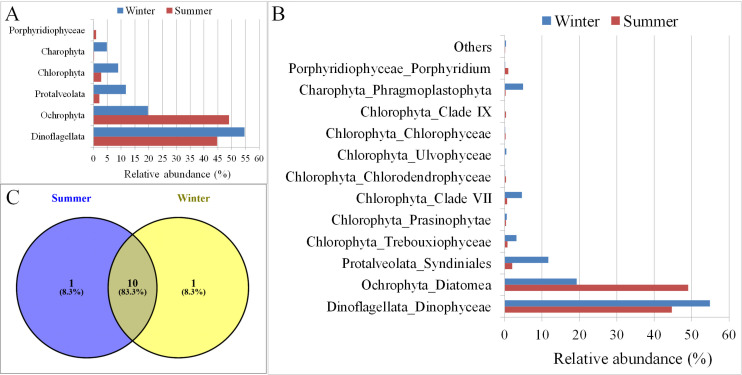
Marine phytoplankton diversity between summer and winter across different sampling stations in Kuwait Waters. A and B: Microalgae groups. C. Shared and unique microalgae groups.

The algal diversity was compared between summer and winter to investigate the effect of different seasons on the algal community: our results showed a seasonal variation in the algal diversity. Overall, all groups were more abundant during the winter than in the summer, except for Ochrophyta. The phylum Ochrophyta was predominantly (~50%) found during the summer season as compared to the winter (~20%) (**Fig 5A)**. These results indicate the overall abundance of the algal community was higher during the winter season than in the summer. The increased abundance during the winter was also observed at the subgroup/class level (**Fig 5B**). Furthermore, the organisms from the class Ulvophyceae were observed only during the winter season, while Clade IX organisms were marginally (<1%) present only during the summer season (**Fig 5C)**.

The results showed a variation in the algal community within each station in both seasons (**Fig 4)**. The organisms in Dinoflagellata were less abundant at Stations K3, K18, and K6 during the winter, whereas at the rest of the stations, their abundance increased during the same season. The phylum Ochrophyta was found to be more abundant during the winter at Stations K3 and K18, whereas, at the remaining stations, its abundance increased during the summer season. Furthermore, the subphylum Protalveolata was abundant during the winter season at Stations K3 and K18 (10.6% vs 1.1% and 44.2% vs 11.5%, respectively), while it increased significantly at Station K6 in winter compared with the summer (16.5% vs. 0.5%). The increase in Protalveolata during the winter was also observed in samples from other stations. However, its overall abundance was much lower (<1%) (**Fig 4A)**. The abundance of the Chlorophyta class Trebouxiophyceae was shown to be higher at Station KA during the winter than in the summer (31.5% vs. 3.7%) (**Fig 4B**). Similarly, the abundance of Clade VII was high at Station K18 during winter than in summer (29.6% vs. 4.4%) (**Fig 4B)**.

### Variations in the algae community due to anthropogenic effects

**Fig 6:**
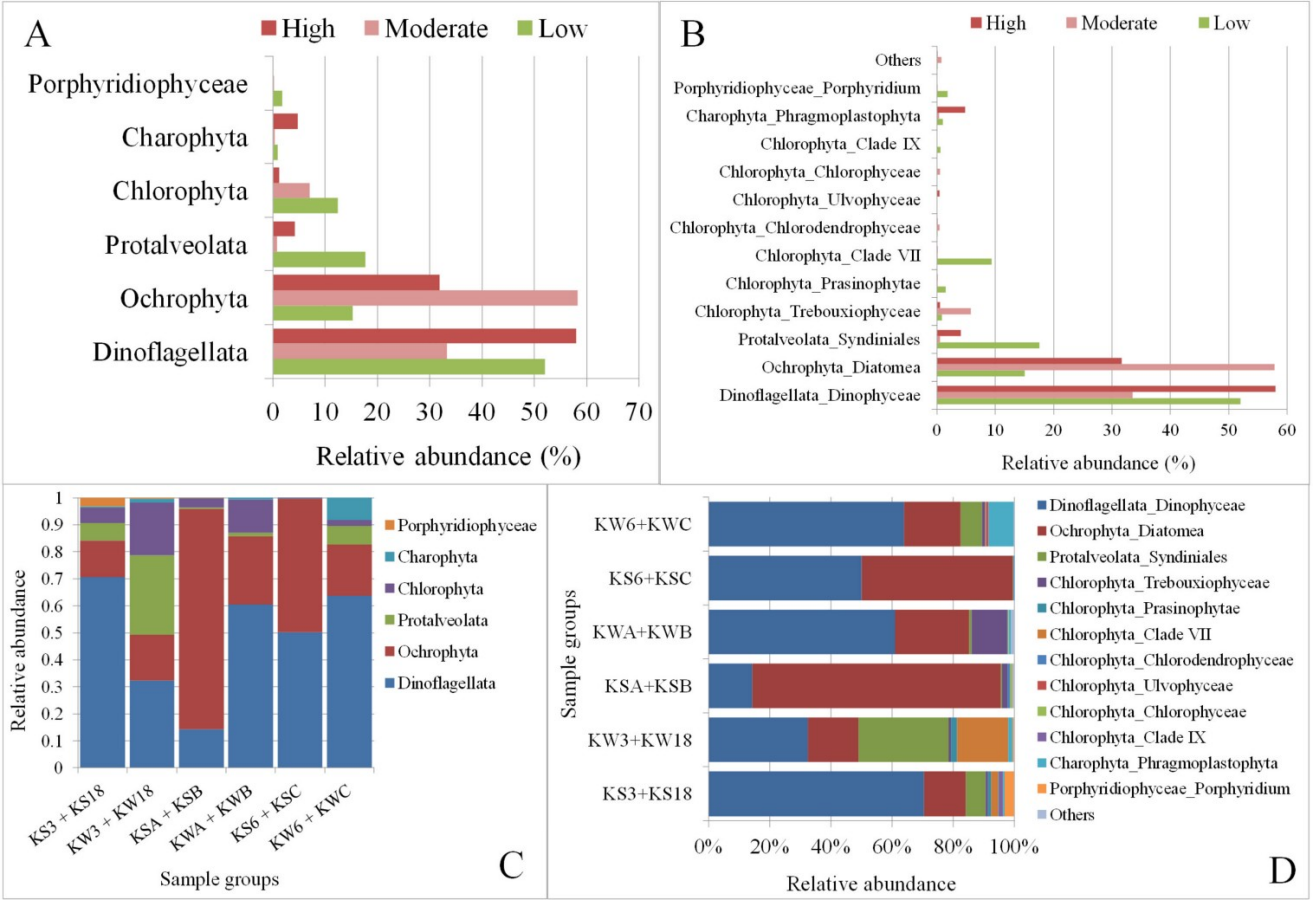
Marine phytoplankton diversity across different sampling stations in Kuwait Bay grouped by degree of anthropogenic activity. A and B: Phytoplankton communities in samples based on anthropogenic activity; C and D: Phytoplankton communities in samples based on anthropogenic activity and season. Descriptions of the sample groups in C & D are as follows: KS3 + KS18: samples collected from stations with low anthropogenic activity in the summer; KW3 + KW18: Samples collected from stations with low anthropogenic activity in the winter; KSA + KSB: Samples collected from stations with moderate anthropogenic activity in the summer; KWA + KWB: Samples collected from stations with moderate anthropogenic activity in the winter; KS6 + KSC: Samples collected from stations with high anthropogenic activity in the summer; KW6 + KWC: Samples collected from stations with high anthropogenic activity in the winter.

We also explored the influence of different degrees of anthropogenic activity on the structure of the algal communities at different stations (**Fig 6)**. Dinoflagellata was most abundant at stations with high levels of anthropogenic activity (**Fig 6A**), with the population being higher in winter than in summer (**Fig 6C**). Furthermore, Ochrophyta was the least abundant at stations with low anthropogenic effects and highest at the station with moderate anthropogenic effects. The higher abundance of Ochrophyta was especially evident during the summer season. The groups Protalveolata and Chlorophyta were the most abundant at the sampling stations with low anthropogenic activity (**Fig 6A)**. Among the Chlorophyta subgroups, Trebouxiophyceae was found to be the most abundant at stations with moderate anthropogenic activity **(Fig 6B)**, and this was mainly observed during the winter (**Fig 6D**). Another Chlorophyta subgroup, Clade VII, was found to be the most abundant in stations with low levels of anthropogenic activity, again mainly during the winter season (**Fig 6D**).

**Fig 7:**
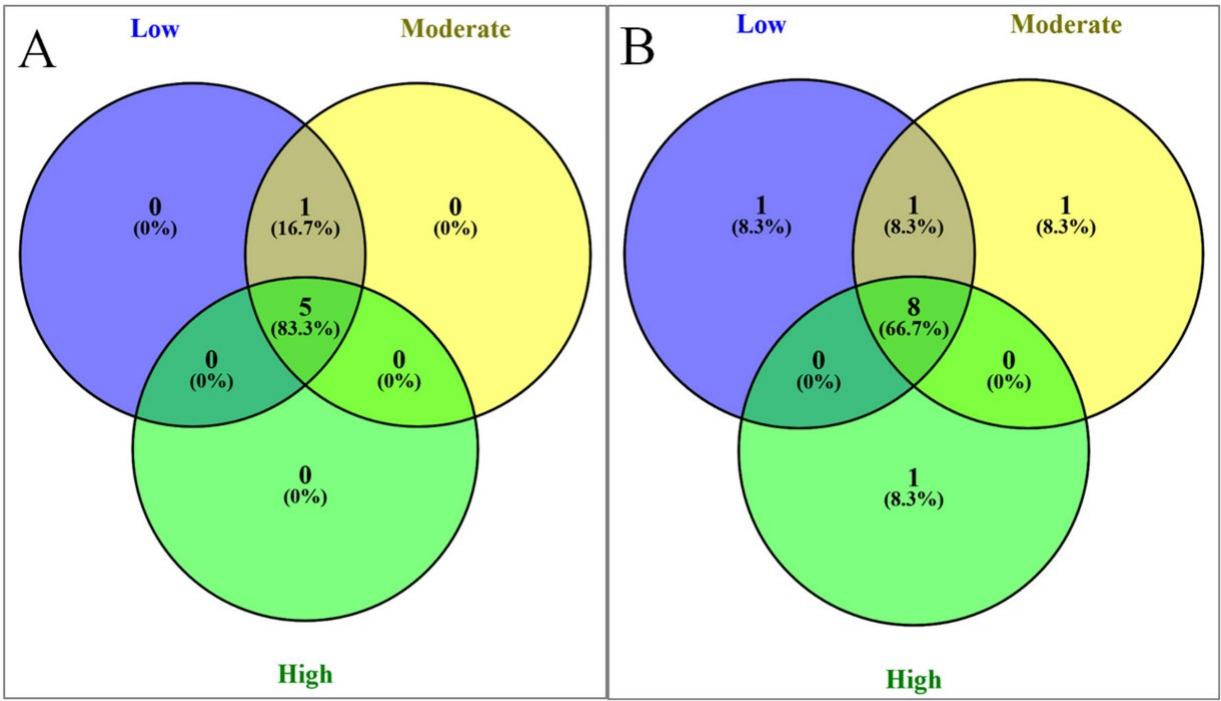
Shared and unique microalgae groups at SILVA classification levels 3 (A) and 4 (B) at stations with varying degrees of anthropogenic effect.

When common and unique taxa were investigated across the locations subject to different levels of anthropogenic effects, Porphyridiophyceae was not detected in locations with high levels of anthropogenic effects (**Fig 7A)**. Among the Chlorophyta subgroups, Ulvophyceae was found to be uniquely present in stations characterized by high anthropogenic effects, whereas Chlorophyceae and Clade IX were found to be present in stations with moderate and low levels of anthropogenic effects, respectively (**Fig 7B)**.

### A comparative account of algal and other eukaryotic microbial communities

**Fig 8:**
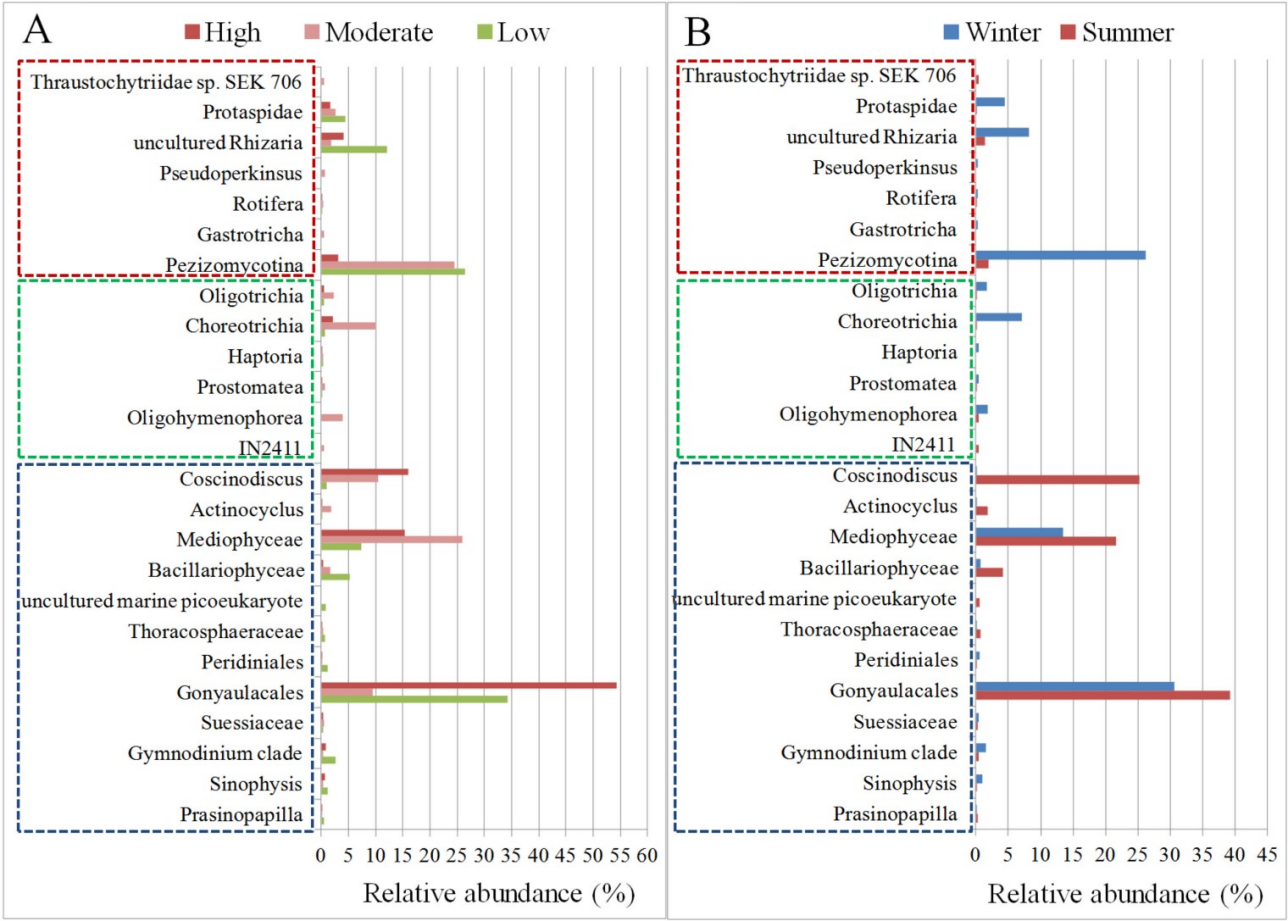
The relative abundance of eukaryotic microbial taxa based on the degree of anthropogenic changes (A) and season (B). Taxa with an overall abundance of >0.1% are shown. The different groups of organisms are marked with different color dotted boxes. Blue dotted box: Algae; Green dotted box: Ciliates; Red dotted box: Fungi, protists, and other microscopic eukaryotes.

The abundances of different eukaryotic microbial communities at different stations in Kuwait waters are shown in **Fig 8 and S3 File**. As expected, algae-related taxa were the most abundant across most of the samples, except for samples KW3, KWA, KWB **(S2 and S3 Figs)**. We also explored the variation in the microbial eukaryotic communities at different stations, based on the degree of anthropogenic activity and the season. The overall abundance of the members of the Dinoflagellata, order Gonyaulacales was comparatively higher in stations with high anthropogenic activity (~55%, *P* < 0.05), irrespective of the season (**Fig 8A**). Their absolute abundance was higher in the summer and lower during the winter season (**Fig 8B and S4 Fig**). Interestingly, the fungal taxa *Pezizomycotina* was highly abundant during the winter season and decreased significantly during the summer (*P* < 0.005) (**Fig 8B)**. This trend was noticed in stations with low or moderate anthropogenic activity. However, in stations with high anthropogenic activity, this fungus was only marginally, albeit significantly, present (*P* < 0.05 in high vs. low and *P* < 0.05 in high vs. moderate) **(S4 Table**). *Coscinodiscus*, a genus of diatoms, was found at significantly higher (*P* <0.00001) levels during the summer than in the winter (**Fig 8B**). It was interesting to note that its abundance also increased in stations that have high levels of anthropogenic activity (**Fig 8A and S4 Table**). Protaspidae, a family of protists, was found to be significantly more abundant during the winter than the summer (*P* < 0.005) at all sampling stations (**Fig 8B, S4 Table**). In addition, although less, its abundance was shown to decrease with high levels of anthropogenic activity (**Fig 8A**). Choreotrichia and Oligotrichia, both ciliates, were found to be significantly more abundant during winter (*P* <0.00001 and *P* < 0.005, respectively) than in the summer (**Fig 8B**, **S4 Table)**, and were more abundant at stations with high or moderate anthropogenic activity (**Fig 8**). Comparison of the eukaryotic microbial communities at stations with different anthropogenic activity levels showed 19 taxa to be in common, whereas one taxon (uncultured marine Picoeukaryote) and five taxa (IN2411, Oligohymenophorea, Gastrotricha, Pseudoperkinsus, Thraustochytriidae sp. SEK 706) were unique to stations with low and moderate degrees of anthropogenic activity, respectively, with an abundance threshold of 0.1%. No unique taxon was found in stations with high degrees of anthropogenic effects (**S5A Fig**). Six taxa found to be seasonally specific: Haptoria, Gastrotricha, and Pseudoperkinsus were recorded solely in the summer, whereas uncultured marine Picoeukaryote, IN2411, and Thraustochytriidae sp. SEK 706 were only present in the winter (**S5B Fig)**.

### Dinoflagellate genus and species in Kuwait Bay

**Fig 9:**
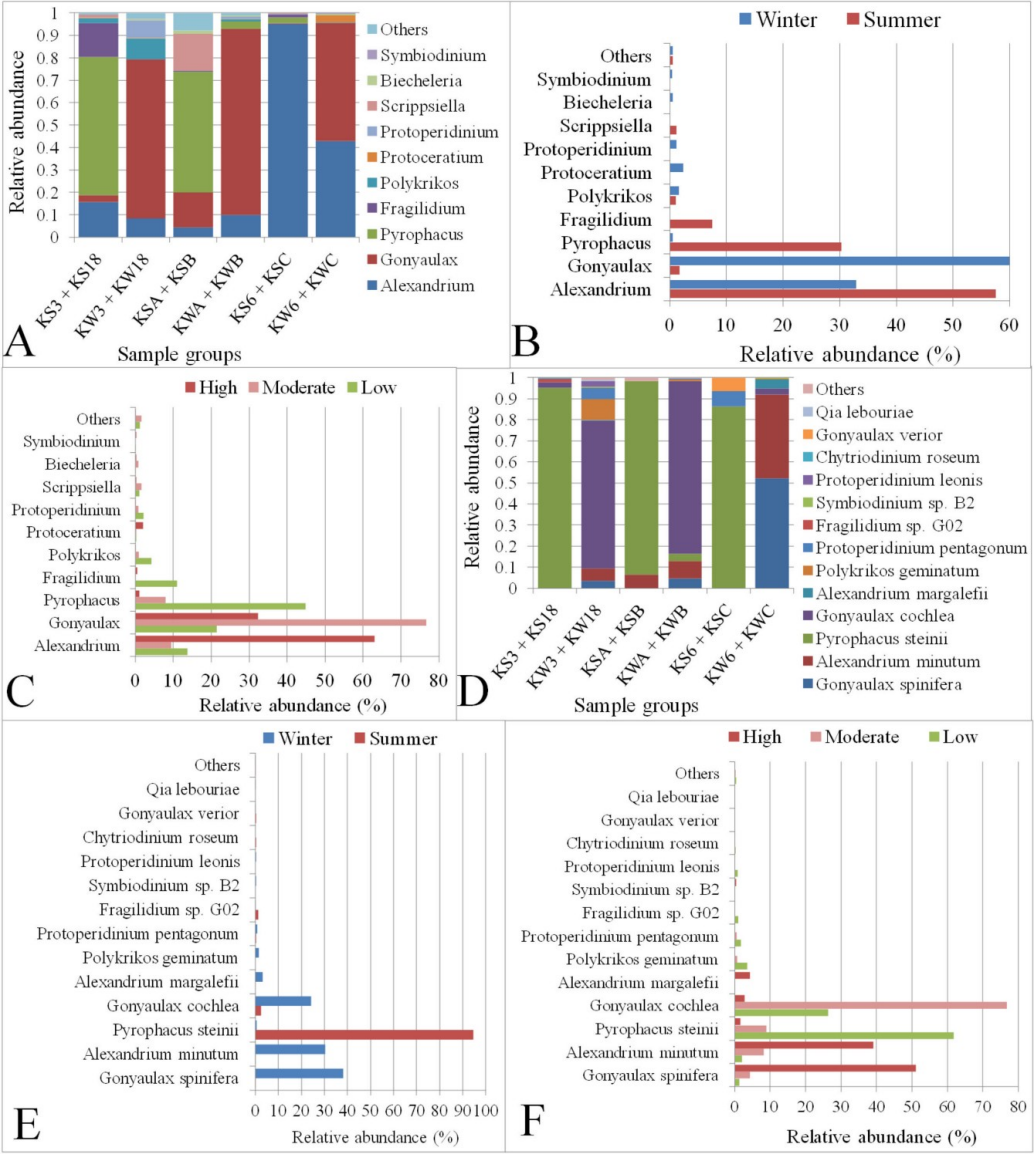
Relative abundance of Dinoflagellata genera and species in Kuwait Bay. A, B and C: Abundances of Dinoflagellata genera in samples grouped based on sampling station, season, and anthropogenic activity level, respectively. D, E and F: Abundances of Dinoflagellata species in samples grouped based on sampling station, season and anthropogenic activity levels, respectively. The term “Others” on the Y-axis includes genera with an abundance of <0.1% in B and C or species <0.05% in E and F. KS3 + KS18: Samples collected in summer from stations with low anthropogenic activity levels; KW3 + KW18: Samples collected in winter from stations with low anthropogenic activity levels; KSA + KSB: Samples collected in summer from stations with moderate anthropogenic activity levels; KWA + KWB: Samples collected in winter from stations with moderate anthropogenic activity levels; KS6 + KSC: Summer-collected samples from stations with high anthropogenic activity levels; KW6 + KWC: Winter-collected samples from stations with high anthropogenic activity levels.

Dinoflagellata was found to be the most abundant taxon among the algal groups present in Kuwait Bay, contributing about 50% of the total eukaryotic microbial abundance. Species related to Dinoflagellata have been reported to be associated with red tides in Kuwait’s waters, as well as with mortality of seabirds and fish [40,41]. Therefore, we examined the various genera and species belonging to the Dinoflagellata phylum at different stations in Kuwait Bay. A total of 28 genera were classified across various locations in Kuwait Bay. Of these, ten genera had an abundance of at least 0.1% (**Fig 9** and **S4 File**): *Alexandrium* was found to be the dominant genus with 44% relative abundance, followed by *Gonyaulax* with a relative abundance of 33.5%. Although the overall abundance of the *Alexandrium* was the highest, it was not the most abundant genera in all samples (**S6 Fig**). We observed a variation in the abundance of Dinoflagellate genera in water samples collected from the same stations during different seasons. For example, *Pyrophacus* was found to be significantly (*P* < 0.0005) abundant in most of the stations during the summer season, whereas it was very low or undetectable during the winter (**Fig 9D, S6 Fig**). Similarly, *Gonyaulax* was significantly (*P* < 0.005) more abundant during the winter (~60%) compared with the summer (~2%) (**Fig 9A** and **9B, S6 Fig** and **S4 File)**. Additionally, a few genera, such as *Pyrophacus*, *Polykrikos*, *Fragilidium* were abundant at stations with minimum anthropogenic activity, whereas *Alexandrium* and *Gonyaulax* were plentiful in stations subject to higher anthropogenic activity. The changing abundance of *Pyrophacus* (*P* < 0.0005) and *Alexandrium* (*P* <0.005) at stations with varying degrees of anthropogenic levels was statistically significant (**Fig 9C** and **S5 File)**.

A total of 44,387 sequences were classified into 22 known Dinoflagellata species. Among these, *Gonyaulax spinifera* was found to be the most abundant (~30%) species, followed by *Alexandrium minutum* (~24%) and *Pyrophacus steinii* (21%), (**S7 Fig** and **S4 File)**. Two species of *Gonyaulax*, *G*. *spinifera*, and *G*. *cochlea*, were abundant in the winter but were either absent or present in low numbers during the summer across all stations (**Fig 9D and 9E)**. However, the relative abundance of *G*. *spinifera* during the winter in comparison to its abundance during the summer was statistically significant (*P* < 0.05) (**S5 File)**. *G*. *spinifera* was observed to be highly abundant at stations characterized by high anthropogenic activity levels, whereas *G*. *cochlea* was more abundant at stations with moderate to low anthropogenic activity. (**Fig 9F)**. Furthermore, *A*. *minutum* was significantly (*P* < 0.005) more abundant in winter than in summer, and was more abundant at stations subject to high levels of anthropogenic effects (**Fig 9D–9F)**. The abundance of *P*. *steinii* was significantly (*P* = 8.01E-06) higher in summer, mainly in stations with low levels of anthropogenic activity (**Fig 9E and 9F)**. The remaining Dinoflagellata species, however, were less abundant across stations, and a few were present exclusively at specific stations (**S4 File)**.

### Network analysis of Dinoflagellata genera

**Fig 10:**
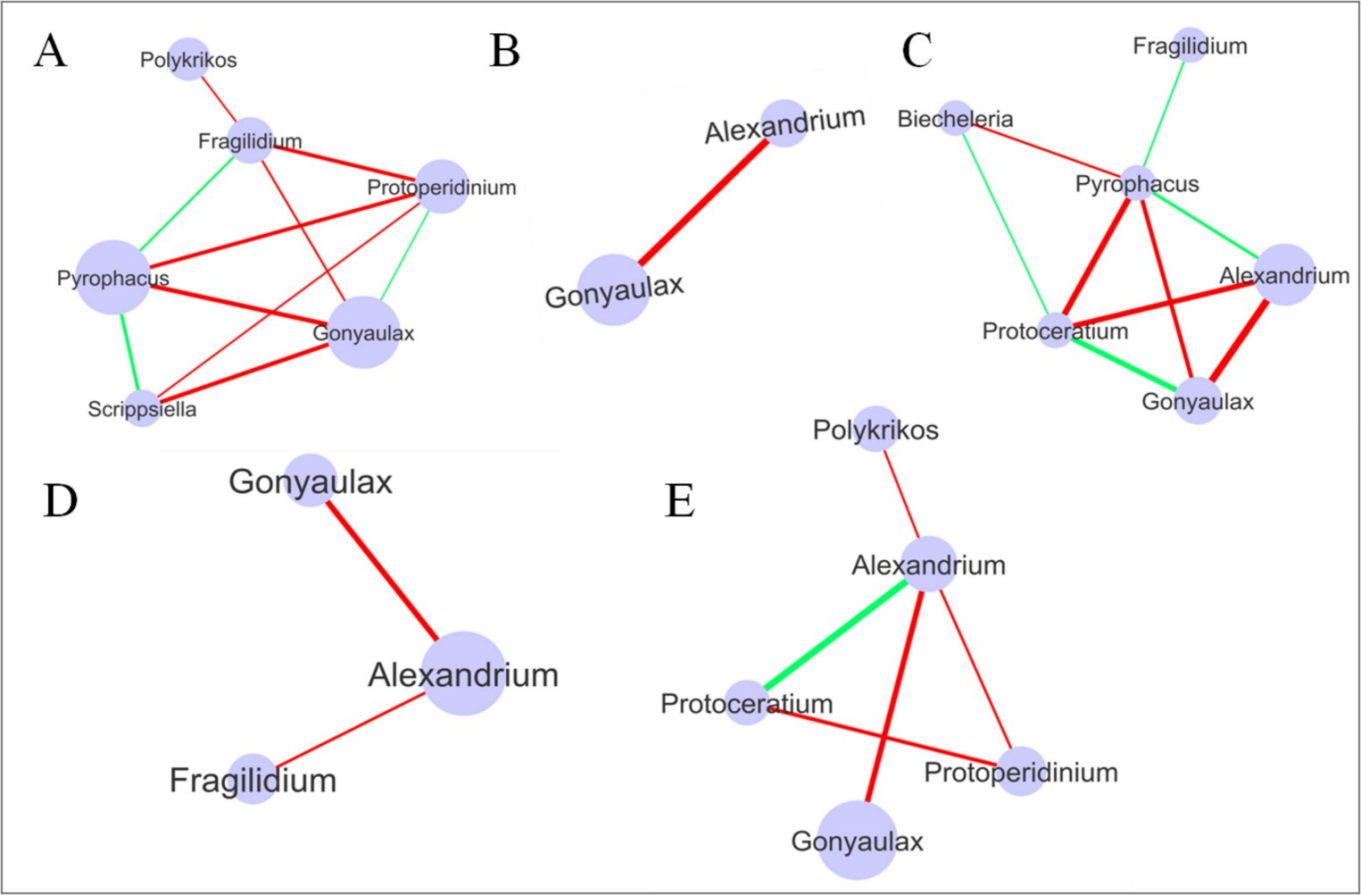
Network analysis of Dinoflagellata genera in stations with varying anthropogenic activity levels and during different seasons. A: Low pollution; B: Moderate pollution; C: High pollution; D: Summer; E: Winter. Node size represents overall relative abundance. The green color line represents co-presence (positive correlation) and the red color line represents mutual exclusion (negative correlation) between the taxa. The line thickness represents the correlation coefficient (a higher correlation coefficient is represented by a thicker line).

The network analysis across stations subject to different degrees of anthropogenic effects, as well as showing seasonal effects, showed comparatively more negative correlations (mutual exclusion) than positive correlations (co-presence) among the Dinoflagellate genera. The results identified more genera that were significantly correlated in the stations with low and high degrees of anthropogenic activity (**Fig 10A–10C**). The stations with low pollution levels had 6 genera connected with 3 positive and 7 negative correlations. *Scrippsiella* and *Pyrophacus* were the most significantly positively correlated (r = 0.701; P < 0.005), whereas *Pyrophacus* and *Gonyaulax* were the most significantly negatively correlated (r = 0.81; P < 0.0005) genera in stations with low pollution (**Fig 10A**). The stations with moderate pollution had 2 genera significantly negatively correlated (r = 1; P = 0) (**Fig 10B**). The stations with a high degree of pollution had 6 genera connected, with 4 positive and 5 negative correlations. Among these, *Gonyaulax* and *Protoceratium* were the most significantly positively correlated (r = 0.82; P < 0.0005), whereas *Alexandrium* and *Gonyaulax* were the most significantly negatively correlated (r = 0.92; P = 3.99E-07) genera (**Fig 10C**).

The network analysis of genera from the samples collected during the two different seasons showed more significantly correlated genera during the winter than during the summer (**Fig 10D and 10E**). During the summer season, *Alexandrium* was negatively correlated with both *Gonyaulax* (corr. = 0.51; P = 0.012) and *Fragilidium* (corr. = 0.42; P = 0.037) (**Fig 10D**). However, 5 genera were connected with 1 positive and 4 negative correlations in the samples collected during the winter. While *Alexandrium* was most significantly positively correlated with *Protoceratium* (r = 0.73; P < 0.0001), it was found to be most significantly negatively correlated with *Gonyaulax* (r = 0.66; P < 0.0008) (**Fig 10E**).

### Diatom genus and species in Kuwait Bay

**Fig 11:**
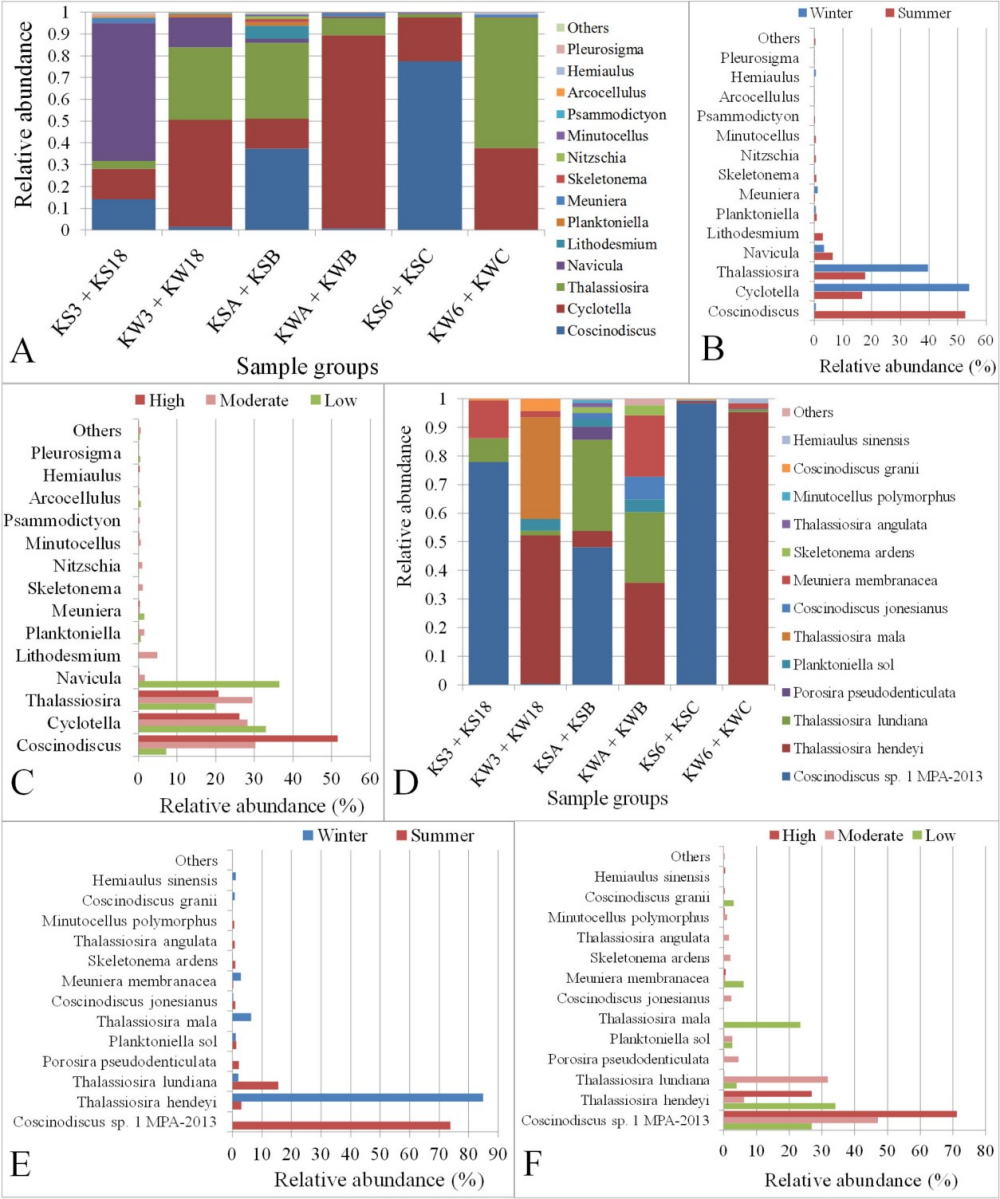
Relative abundance of Diatom genera and species in Kuwait water. A, B and C: Abundances of Diatom genera in samples grouped based on sampling station, season and anthropogenic activity level, respectively. D, E and F: Abundances of Diatom species in samples grouped based on sampling station, season and anthropogenic activity level, respectively. The term “Others” in the Y-axis includes genera or species with an abundance of <0.1%. KS3 + KS18: Samples collected in summer from stations with low anthropogenic activity levels; KW3 + KW18: Samples collected in winter from stations with low anthropogenic activity levels; KSA + KSB: Samples collected in summer from stations with moderate anthropogenic activity levels; KWA + KWB: Samples collected in winter from stations with moderate anthropogenic activity levels KS6 + KSC: Samples collected in summer from stations with high anthropogenic activity levels; KW6 + KWC: Samples collected in winter from stations with high anthropogenic activity levels.

Diatoms were the second most abundant taxon among the algal groups present in Kuwait Bay with an overall abundance of 34%. A total of 24 diatom genera were classified across different locations in Kuwait waters (**S6 File**), among which 14 genera had an abundance of at least 0.1% (**S8 Fig**). Five diatom genera, *Coscinodiscus*, *Cyclotella*, *Thalassiosira*, *Navicula*, and *Lithodesmium*, covered 97% of all diatoms in Kuwait Bay. *Coscinodiscus* was the most dominant genus, with an abundance of 37% followed by *Cyclotella* (28%) among the classified diatom genera. The high abundance of *Coscinodiscus* was observed in the water samples collected during the summer season (*P* = 0.0006) from different stations (**Fig 11A and 11B**). Similarly, the diatom genus *Lithodesmium* was present only during the summer season and not in the winter (*P* = 2.21E-07). However, *Thalassiosira* and *Cyclotella* were highly abundant in the winter season, although they were statistically insignificant (**Fig 11B**). Furthermore, *Coscinodiscus* abundance increased with the level of anthropogenic activity (**Fig 11C**), although the increase in abundance was statistically insignificant. In contrast, the genus *Navicula* was present in high abundance at stations (Station KSA and KS18) with less anthropogenic activity (**Fig 11A and 11C**).

Among the diatom species, *Coscinodiscus sp*. *1 MPA-2013*, *Thalassiosira hendeyi*, *Thalassiosira lundiana*, *Porosira pseudodenticulata*, *Planktoniella sol*, and *Thalassiosira mala* covered 96% of the total abundance (**S6 File** and **S9 Fig**). *C*. *sp*. *1 MPA-2013* was the dominant species with an abundance of 60% (**Fig 11D**). Furthermore, a comparison of species observed during the summer and the winter indicated that *C*. *sp*. *1 MPA-2013* was significantly more abundant in summer than in winter (74%, *P* = 3.14E-08), (**S7 File).** However, *Thalassiosira hendeyi* and *T*. *lundiana* were more abundant in the winter and the summer, respectively (**Fig 11E)**. The species, *Porosira pseudodenticulata*, *T*. *angulata*, and *Minutocellus polymorphus* were found exclusively during the summer season, whereas *T*. *mala* and *Hemiaulus sinensis* were exclusively found in the winter season. Additionally, *C*. *sp*. *1 MPA-2013* showed an increase in abundance with an increasing level of anthropogenic effects, whereas *Thalassiosira mala* was only present in stations that were not prone to anthropogenic disturbances and farther away from the coast (**Fig 11D and 11F**).

## Discussion

The taxonomy and distribution of phytoplankton species in Kuwait waters have been studied for over a decade [23,26,42–46]. The studies were focused on periodic water and sediment sampling from well-defined sampling stations across Kuwait waters. These community studies were performed using microscopy-based identification and statistical analysis. Molecular techniques are considered more advanced and complement conventional taxonomy techniques for taxonomic identification and community analysis of environmental samples because of their high-throughput nature, which allows analysis of multiple samples at a time.

As per our knowledge, there are no published reports on NGS-based microbiome studies in relation to anthropogenic activity in this region. The data pertaining to the microbial taxa and its association with the degree of anthropogenic activity is tentative as we did not perform real-time analysis of the water quality parameters during the study period. However, we have grouped the sampling stations into three categories of anthropogenic activity levels (high, moderate, and low) based on the information and data obtained from various published papers.

The probable sources of high anthropogenic activity can be attributed to the introduction of effluents from various industries, petrochemical and power plants, high salinity near desalination plants, and other point sources of pollution through the occasional discharge of raw sewage from emergency and storm outlets discharge. Petroleum hydrocarbons, trace metals, suspended particles, and nutrients from the above-mentioned sources adversely affect the water quality in stations K6 and KC (designated as stations K6 and C in previous publications [24,25,30,47,48]). A high phosphate and nitrate level is mainly due to the discharge of desalination, power plant effluences and sewage. It is also worth noting that, at certain instances, the levels reached up to 349.31 μg/l of NO_3_ and 1434.3 μg/l of PO_4_ [24]. The Shatt Al Arab river’s water discharge is a vital nutrient influx source in the northern part of Kuwait bay that generates a nutrient level gradient from north to south in Kuwait bay. The mean concentration of phosphate and nitrate were 14.9 μg/l and 52.5 μg/l respectively [24]. An alarmingly high level of total organic carbon (TOC) was also observed in Kuwait bay [25] indicating that, the high TOC may be directly related to the petroleum hydrocarbon pollution in surface waters. Stations KA and KB are situated in zones near the point sources of pollution and experience a moderate degree of anthropogenic activities. Stations K3 and K18 are situated offshore away from most point sources of pollution.

Our microbiome analysis results confirmed the presence of many eukaryotic communities, including algae, fungi, flagellates, amoeboids, and protists in the water samples. The rarefaction plot affirmed the sequencing depth that is adequate to cover the eukaryotic taxa in the water samples (**S1 Fig**). Furthermore, the clustering of the seasonally collected samples indicates a similar eukaryotic community across different sampling stations during the same season, with a few exceptions. Among the eukaryotic groups, SAR, Archaeplastida, and Opisthokonta together covered 99.8% of the sequences. Among these, the SAR group was found to be the most abundant having 61.4% coverage. Multiple studies have shown a high abundance of SAR groups in seawater [49,50]. In the SAR super-group, Alveolata was the most abundant super-kingdom followed by Stramenopiles and Rhizaria with overall abundances of 52.3%, 39.2%, and 8.5%, respectively. These data are in line with those observed in the South Sea (Tongyeong coast) of South Korea [49].

Our results showed a clear indication of a change in the algal community based on season and possible anthropogenic activity. Dinoflagellates showed a higher abundance in the winter season, whereas diatoms were more abundant in the summer season. The findings were similar to those of Devlin et al. (2019), who analyzed phytoplankton data for 10 years, and found higher abundances of dinoflagellates in February and of diatoms in June and September in Kuwait Bay [51]. Furthermore, they also reported that the abundance of dinoflagellates was higher in Kuwait Bay than in the Arabian Gulf. The alpha diversity and richness indices of the samples collected from the stations with a probability of high anthropogenic activity levels were lower than those of stations with a low to moderate degree of anthropogenic activity. The possible reasons for the decrease in phytoplankton diversity in these stations could be attributed to increased nutrient load, salinity [23] and possibly due to an overall high degree of anthropogenic activities in Kuwait Bay.

Among the algal groups, the phyla Dinoflagellata and Ochrophyta (phyla containing diatoms) together had an abundance of 84%. Hence, we further studied the genus and species related to Dinoflagellata and Ochrophyta. In the current study, the Dinoflagellata genera *Alexandrium*, and *Gonyaulax* were abundant in the water samples collected from stations with high exposure to anthropogenic activity. Furthermore, *Alexandrium* was dominant during summer whereas *Gonyaulax* was predominant during the winter season. *Alexandrium* produces toxic harmful algal blooms (HAB), which cause paralytic shellfish poisoning in humans. The effects of toxins produced by *Alexandrium* species on marine bivalves are well documented. In many regions, incidences of blooms caused by *Alexandrium minutum* during the reproduction season of bivalves, such as *Crassostrea gigas*, have been reported. These blooms affect gametogenesis, spawning, and larval development [52]. Broodstock exposed to *A*. *minutum* showed reduced motility of spermatozoa, reduced larval size, and increased mortality during settlement, which indicates the effect of *A*. *minutum* blooms during gametogenesis, spawning or larval development. Episodes of massive fish kills occurred in Kuwait during various instances. Investigations carried out during the 1999 fish kill were attributed to a bloom of the dinoflagellates *Gymnodinium* sp. The cell count showed a staggeringly high number, exceeding 6 x 10^6^ cells/ml in the area of fish kills, contributing it as a major cause for the fish kill. Heil et al (2001) have reported 27 diatom and 21 dinoflagellate species, which were present in abundant numbers during the bloom event [40]. In a subsequent event of the massive fish kill, the red tide patch showed a greatly increased abundance of *Alexandrium minutum*, *Gymnodinium catenatum*, and *Gyrodinium impudicum* [40,41].

Different species of *Alexandrium* have been reported in the waters of Japan [53], Chile [54], New Zealand [55], United States [56], and other parts of the world [57–59]. The species of the *Alexandrium* genus cause the death of shellfish and poisoning to humans who consume affected shellfish. We observed a high abundance of *Alexandrium minutum* in stations K6 and KC during the winter season (Fig 9). Stations K6 and KC are in Kuwait Bay and more prone to anthropogenic activities. Coquereau et al. (2016) recorded alterations in the valve movements of the great scallop, *Pecten maximu*,*s* upon exposure to *A*. *minutum* [60]. Another Dinoflagellate genus, *Gonyaulax*, is known to produce yessotoxins associated with toxic algal blooms [61]. In the current study, *Gonyaulax spinifera* and *A*. *minutum* both showed a similar trend of being abundant in winter seasons in stations K6 and KC, which are the stations prone to high anthropogenic activities in Kuwait Bay. In contrast, the Dinoflagellate genus *Pyrophacus* was predominant during the summer. This is consistent with a similar observation by Liang et al, who found an increased *Pyrophacus* in the northern Yellow Sea during the summer season [62].

The second most dominant microalgae group in Kuwait Bay was the diatom (34%). The *Coscinodiscus* genus consisted of *C*. *sp*. *1 MPA-2013*, *C*. *jonesianus*, and *C*. *granii*. They were observed in stations with high and moderate anthropogenic activities during summer. A study conducted in the Arabian Ocean in India showed an increase in the abundance of diatoms, including *Coscinodiscus*, during the summer months at locations where petroleum hydrocarbon waste was discharged into the ocean [63]. Kuwait bay is a nutrient rich, productive coastal area with high levels of hydrocarbons discharged to the bay from both petrogenic and anthropogenic sources. Saeed et al. (2018) reported 3.6 μg/kg—up to 20,030 μg/kg of polycyclic aromatic hydrocarbons (PAHs) in Kuwait Bay [48]. We believe that the high abundance of *Coscinodiscus* and *Alexandrium* sp is mainly due to the high anthropogenic activity in stations in K6 and KC. In contrast, the genus *Navicula* was most abundant at stations with low levels of anthropogenic activity (stations K3 and K18). A few species of *Navicula*, such as *N*. *atomus* and *N*. *cryptocephala*, have been reported to be resistant to organic pollution [64], whereas some others, such as *N*. *lanceolate*, are sensitive to pollution. Furthermore, the species of the diatom genera *Nitzschia* and *Cyclotella* are known to be tolerant to organic pollution and are abundant in heavily and moderately polluted sites in some rivers of Vietnam [65].

Tromas et al. (2017) were able to predict the onset of a cynobacterial bloom with high accuracy using 16S rRNA sequencing. They studied the bacterial community in a eutrophic lake over time to understand the repeatability of cyanobacterial blooms and found that high throughput sequence data were an excellent predictor of the onset of a bloom [66]. A number of studies indicate that the increase in incidents of algal blooms is the result of increased sea surface temperature and changes in nutrient loads and ratios [59,67–72]. These studies stress the importance of understanding the basis of recurrent episodes of fish kills and bivalve mortality and their association with episodes of algal blooms in Kuwait. The frequent episodes of fish kill and association of algal blooms highlight the need for periodic monitoring using traditional and advanced molecular tools to document the changes in the population dynamics of phytoplankton in Kuwait waters.

## Conclusions

Our study shows the successful employment of 18S rRNA gene sequencing to elucidate the structure of the phytoplankton community at different locations in Kuwait waters in two different seasons. The current study, furthermore, explored the possible link between anthropogenic activity and variation in the phytoplankton community. Our results confirmed the differential abundance of dinoflagellates and diatoms, at the selected sampling stations and the seasons. The findings from the current study can form baseline data for future community ecology studies. Also, this approach can be used to assess the possible link between water quality and the differential abundance of specific microalgae, such as *Alexandrium*, *Pyrophacus*, *Coscinodiscus*, and *Navicula*. The NGS approach along with water quality analysis in parallel could be further extended to identify indicator species associated with water pollution. We speculate that advancements in NGS and real-time assessment of various parameters in the marine environments will enable greater opportunities for the regulatory agencies to monitor the ecosystem. Integration of NGS-based biological data and water analysis with artificial intelligence and machine learning techniques will further offer powerful real-time data analysis tools for better monitoring and early prediction of algal blooms in the marine ecosystem, potentially allowing time for the implementation of appropriate mitigation measures.

## Supporting information

S1 Fig Shannon rarefaction plot. (DOC)

S2 Fig Relative abundance of eukaryotic microbial taxa across all samples. Taxa with an overall abundance of >0.1% are shown. The different group of organisms are marked with different color dotted boxes. Blue dotted box: Algae; Green dotted box: Ciliates; Red dotted box: Fungi, protist, and other microscopic eukaryotes.
(DOCX)

S3 Fig Relative abundance of eukaryotic microbial taxa across all sample groups. Taxa with an overall abundance at least 0.1% are shown. The different group of organisms are marked with different color dotted boxes. Blue dotted box: Algae; Green dotted box: Ciliates; Red dotted box: Fungi, protist, and other microscopic eukaryotes.
(DOCX)

S4 Fig Relative abundance of eukaryotic microbial taxa in stations with varying anthropogenic activity and different season. Taxa with an overall abundance of >0.1% are shown. The different group of organisms are marked with different color dotted boxes. Blue dotted box: Algae; Green dotted box: Ciliates; Red dotted box: Fungi, protist, and other microscopic eukaryotes.
(DOCX)

S5 Fig Shared and unique eukaryotic microbial communities across stations with different anthropogenic activity (A) and between seasons (B).
(DOCX)

S6 Fig Relative abundance of Dinoflagellate genera across all samples collected from different sampling stations of Kuwait Bay. ‘Others’ include genera with an abundance of <0.1%.
(DOCX)

S7 Fig Relative abundance of Dinoflagellate species across samples collected from different sampling stations of Kuwait Bay. ‘Others’ include genera with an abundance of <0.05%.
(DOCX)

S8 Fig Relative abundance of diatom genera across all samples collected from different sampling stations of Kuwait Bay. ‘Others’ include genera with an abundance of <0.1%.
(DOCX)

S9 Fig Relative abundance of diatom species across all samples collected from different sampling stations of Kuwait Bay. ‘Others’ include genera with an abundance of <0.1%.
(DOCX)

S1 Table Summary of data after various filtering steps. (DOCX)

S2 Table Alpha diversity indices for individual samples. (DOCX)

S3 Table Pairwise comparison of alpha diversity indices of same stations between summer and winter seasons. (DOCX)

S4 Table Differential abundance *P* value of eukaryotic microbial taxa in different comparisons. (DOCX)

S1 File Eukaryotes level1. (XLS)

S2 File AlgalGroups level3. (XLS)

S3 File Eukaryote level6. (XLS)

S4 File Eukaryotes Dinoflagellata. (XLS)

S5 File Significant Dinoflagellata genus-sp. (XLS)

S6 File Eukaryotes diatom. (XLS)

S7 File Diatom DiffSig genus-species. (XLS)
