# Supplementary material for: Distribution and diversity of eukaryotic microalgae in Kuwait waters assessed using 18S rRNA gene sequencing
Source: PLoS One. 2021 Apr 26;16(4):e0250645. doi: 10.1371/journal.pone.0250645 (PMC8075240; doi:10.1371/journal.pone.0250645)
Supplement: S1 Fig — (DOC) [file pone.0250645.s001.doc]

**Distribution and Diversity of Eukaryotic Microalgae in Kuwait Waters Assessed Using 18S rRNA Gene Sequencing**

**Vinod Kumar*, Sabah AlMomin, Vanitha V Kumar, Jasim Ahmed, Lamya Al-Musallam, Anisha B Shajan, Hamed Al-Aqeel, Hamad Al-Mansour, Walid M Al-Zakri**

Environment and Life Sciences Research Center, Kuwait Institute for Scientific Research, Kuwait.

*Corresponding author

Email: [vinodk@kisr.edu.kw](mailto:vinodk@kisr.edu.kw)

Short title: Eukaryotic Microalgae Diversity in Kuwait Waters

**Supplementary Tables and Figures**

Supplementary Figure 1: Shannon rarefaction plot
