# Supplementary material for: Distribution and diversity of eukaryotic microalgae in Kuwait waters assessed using 18S rRNA gene sequencing
Source: PLoS One. 2021 Apr 26;16(4):e0250645. doi: 10.1371/journal.pone.0250645 (PMC8075240; doi:10.1371/journal.pone.0250645)
Supplement: S3 Fig — Taxa with an overall abundance at least 0.1% are shown. The different group of organisms are marked with different color dotted boxes. Blue dotted box: Algae; Green dotted box: Ciliates; Red dotted box: Fungi, protist, and other microscopic eukaryotes. (DOCX) [file pone.0250645.s003.docx]

Supplementary Figure 3: Relative abundance of eukaryotic microbial taxa across all sample groups. Taxa with an overall abundance at least 0.1% are shown. The different group of organisms are marked with different color dotted boxes. Blue dotted box: Algae; Green dotted box: Ciliates; Red dotted box: Fungi, protist, and other microscopic eukaryotes.
