# Supplementary material for: Distribution and diversity of eukaryotic microalgae in Kuwait waters assessed using 18S rRNA gene sequencing
Source: PLoS One. 2021 Apr 26;16(4):e0250645. doi: 10.1371/journal.pone.0250645 (PMC8075240; doi:10.1371/journal.pone.0250645)
Supplement: S5 Fig — Shared and unique eukaryotic microbial communities across stations with different anthropogenic activity (A) and between seasons (B). (DOCX) [file pone.0250645.s005.docx]

Supplementary Figure 5: Shared and unique eukaryotic microbial communities across stations with different anthropogenic activity (A) and between seasons (B).
