# Supplementary material for: Distribution and diversity of eukaryotic microalgae in Kuwait waters assessed using 18S rRNA gene sequencing
Source: PLoS One. 2021 Apr 26;16(4):e0250645. doi: 10.1371/journal.pone.0250645 (PMC8075240; doi:10.1371/journal.pone.0250645)
Supplement: S6 Fig — ‘Others’ include genera with an abundance of <0.1%. (DOCX) [file pone.0250645.s006.docx]

Supplementary Figure 6: Relative abundance of Dinoflagellate genera across all samples collected from different sampling stations of Kuwait bay. ‘Others’ include genera with an abundance of <0.1%.
