# Supplementary material for: Distribution and diversity of eukaryotic microalgae in Kuwait waters assessed using 18S rRNA gene sequencing
Source: PLoS One. 2021 Apr 26;16(4):e0250645. doi: 10.1371/journal.pone.0250645 (PMC8075240; doi:10.1371/journal.pone.0250645)
Supplement: S1 Table — (DOCX) [file pone.0250645.s010.docx]

**Supplementary Table 1: Summary of data after various filtering steps**

| **Sample ID** | **Raw reads** | **Filtered reads** | **Denoised reads** | **Merged sequences** | **Non chimeric sequences** | **% of non chimeric sequences** |
| --- | --- | --- | --- | --- | --- | --- |
| **KS3_1** | 12,455 | 12,195 | 12,020 | 10,427 | 7,877 | 63.2 |
| **KS3_2** | 12,476 | 12,181 | 11,962 | 10,490 | 8,110 | 65 |
| **KS3_3** | 12,555 | 12,286 | 12,110 | 10,672 | 8,067 | 64.3 |
| **KS18_1** | 12,359 | 12,015 | 11,964 | 11,731 | 10,588 | 85.7 |
| **KS18_2** | 12,335 | 12,071 | 11,944 | 11,233 | 10,032 | 81.3 |
| **KS18_3** | 12,436 | 12,153 | 12,055 | 11,526 | 9,677 | 77.8 |
| **KW3_1** | 12,537 | 12,213 | 12,059 | 11,050 | 9,219 | 73.5 |
| **KW3_2** | 12,355 | 12,065 | 11,843 | 11,290 | 10,562 | 85.5 |
| **KW3_3** | 12,381 | 12,020 | 11,909 | 11,421 | 10,893 | 88 |
| **KW18_1** | 12,397 | 12,064 | 11,919 | 11,332 | 9,970 | 80.4 |
| **KW18_2** | 12,494 | 12,202 | 12,076 | 11,439 | 10,434 | 83.5 |
| **KW18_3** | 12,595 | 12,258 | 12,063 | 11,242 | 9,967 | 79.1 |
| **KSA_1** | 12,400 | 12,054 | 11,742 | 10,594 | 8,550 | 69 |
| **KSA_2** | 12,462 | 12,100 | 11,794 | 10,744 | 8,780 | 70.5 |
| **KSA_3** | 12,316 | 12,029 | 11,689 | 10,209 | 7,769 | 63.1 |
| **KSB_1** | 12,454 | 12,148 | 11,971 | 10,708 | 9,651 | 77.5 |
| **KSB_2** | 12,319 | 11,922 | 11,790 | 10,975 | 8,878 | 72.1 |
| **KSB_3** | 12,353 | 12,094 | 11,946 | 10,846 | 8,814 | 71.4 |
| **KWA_1** | 12,498 | 12,189 | 11,859 | 10,842 | 9,559 | 76.5 |
| **KWA_2** | 12,598 | 12,293 | 11,949 | 11,249 | 10,222 | 81.1 |
| **KWA_3** | 12,497 | 12,178 | 11,956 | 11,408 | 11,023 | 88.2 |
| **KWB_1** | 12,398 | 12,062 | 11,825 | 10,731 | 9,103 | 73.4 |
| **KWB_2** | 12,576 | 12,257 | 12,035 | 10,983 | 9,897 | 78.7 |
| **KWB_3** | 12,421 | 12,046 | 11,739 | 10,611 | 9,079 | 73.1 |
| **KS6_1** | 12,474 | 12,169 | 12,032 | 11,102 | 8,497 | 68.1 |
| **KS6_2** | 12,540 | 12,153 | 11,948 | 10,845 | 8,658 | 69 |
| **KS6_3** | 12,540 | 12,167 | 11,974 | 10,761 | 8,868 | 70.7 |
| **KSC_1** | 12,313 | 12,043 | 11,931 | 11,549 | 10,233 | 83.1 |
| **KSC_2** | 12,355 | 12,068 | 11,979 | 11,440 | 10,209 | 82.6 |
| **KSC_3** | 12,333 | 12,085 | 11,998 | 11,474 | 10,437 | 84.6 |
| **KW6_1** | 12,577 | 12,255 | 12,183 | 11,886 | 11,586 | 92.1 |
| **KW6_2** | 12,400 | 12,007 | 11,869 | 11,359 | 10,838 | 87.4 |
| **KW6_3** | 12,615 | 12,296 | 12,139 | 11,384 | 10,768 | 85.4 |
| **KWC_1** | 12,348 | 12,188 | 12,006 | 11,199 | 8,699 | 70.4 |
| **KWC_2** | 12,613 | 12,366 | 12,265 | 11,448 | 9,115 | 72.3 |
| **KWC_3** | 12,409 | 12,194 | 12,073 | 11,361 | 8,810 | 71 |
| **Total** | **448,184** | **437,086** | **430,616** | **399,561** | **343,439** | **76.6** |
