# Supplementary material for: Distribution and diversity of eukaryotic microalgae in Kuwait waters assessed using 18S rRNA gene sequencing
Source: PLoS One. 2021 Apr 26;16(4):e0250645. doi: 10.1371/journal.pone.0250645 (PMC8075240; doi:10.1371/journal.pone.0250645)
Supplement: S2 Table — (DOCX) [file pone.0250645.s011.docx]

**Supplementary Table 2: Alpha diversity indices for individual samples**

| **Samples** | **Shannon index** | **Faith’s PD** | **Pielou’s evenness** |
| --- | --- | --- | --- |
| **KS3_1** | 4.7 | 7.0 | 0.8 |
| **KS3_2** | 5.2 | 6.7 | 0.8 |
| **KS3_3** | 5.2 | 7.1 | 0.8 |
| **KS18_1** | 4.2 | 10.5 | 0.7 |
| **KS18_2** | 5.2 | 13.2 | 0.8 |
| **KS18_3** | 5.3 | 12.2 | 0.8 |
| **KW3_1** | 4.9 | 10.2 | 0.7 |
| **KW3_2** | 5.3 | 14.9 | 0.8 |
| **KW3_3** | 4.5 | 11.7 | 0.7 |
| **KW18_1** | 4.5 | 10.1 | 0.7 |
| **KW18_2** | 4.3 | 10.1 | 0.7 |
| **KW18_3** | 4.3 | 12.3 | 0.7 |
| **KSA_1** | 4.5 | 11.1 | 0.7 |
| **KSA_2** | 4.1 | 12.0 | 0.6 |
| **KSA_3** | 4.8 | 10.6 | 0.7 |
| **KSB_1** | 4.9 | 8.6 | 0.8 |
| **KSB_2** | 4.5 | 9.8 | 0.7 |
| **KSB_3** | 5.0 | 11.0 | 0.8 |
| **KWA_1** | 5.5 | 15.5 | 0.8 |
| **KWA_2** | 4.7 | 16.2 | 0.7 |
| **KWA_3** | 5.2 | 28.4 | 0.7 |
| **KWB_1** | 5.1 | 10.3 | 0.8 |
| **KWB_2** | 5.7 | 9.8 | 0.9 |
| **KWB_3** | 5.4 | 10.6 | 0.8 |
| **KS6_1** | 3.9 | 5.1 | 0.7 |
| **KS6_2** | 4.3 | 6.0 | 0.7 |
| **KS6_3** | 4.3 | 6.6 | 0.7 |
| **KSC_1** | 3.5 | 5.3 | 0.7 |
| **KSC_2** | 3.7 | 6.0 | 0.6 |
| **KSC_3** | 3.3 | 6.3 | 0.6 |
| **KW6_1** | 4.4 | 9.3 | 0.7 |
| **KW6_2** | 5.0 | 11.6 | 0.7 |
| **KW6_3** | 4.8 | 11.1 | 0.7 |
| **KWC_1** | 3.5 | 4.8 | 0.6 |
| **KWC_2** | 3.4 | 4.5 | 0.6 |
| **KWC_3** | 3.3 | 4.1 | 0.6 |
