# Supplementary material for: Distribution and diversity of eukaryotic microalgae in Kuwait waters assessed using 18S rRNA gene sequencing
Source: PLoS One. 2021 Apr 26;16(4):e0250645. doi: 10.1371/journal.pone.0250645 (PMC8075240; doi:10.1371/journal.pone.0250645)
Supplement: S3 Table — (DOCX) [file pone.0250645.s012.docx]

Supplementary Table 3: Pairwise comparison of alpha diversity indices of same stations between summer and winter seasons

| **Sample groups** | | **P value** | | |
| --- | --- | --- | --- | --- |
| **Group 1** | **Group 2** | **Shannon** | **Faith’s PD** | **Pielou’s evenness** |
| KS18 | KW18 | 0.513 | 0.275 | 0.513 |
| KS3 | KW3 | 0.827 | 0.050 | 0.050 |
| KS6 | KW6 | 0.050 | 0.050 | 0.513 |
| KSA | KWA | 0.127 | 0.050 | 0.513 |
| KSB | KWB | 0.050 | 0.513 | 0.050 |
| KSC | KWC | 0.513 | 0.050 | 0.513 |

Statistically significant (Kruskal-Wallis test) values are highlighted in bold font.
