# Supplementary material for: Distribution and diversity of eukaryotic microalgae in Kuwait waters assessed using 18S rRNA gene sequencing
Source: PLoS One. 2021 Apr 26;16(4):e0250645. doi: 10.1371/journal.pone.0250645 (PMC8075240; doi:10.1371/journal.pone.0250645)
Supplement: S4 Table — (DOCX) [file pone.0250645.s013.docx]

Supplementary Table 4: Differential abundance *P* value of eukaryotic microbial taxa in different comparisons

| **Eukaryotic taxa (SILVA level D6)** | **Summer vs. Winter** | **High vs. Low** | **High vs. Moderate** | **Moderate vs. Low** |
| --- | --- | --- | --- | --- |
| uncultured marine picoeukaryote | **1.12E-08** | **2.53512E-06** | 1 | **1.93236E-06** |
| Haptoria | **1.54E-07** | **0.785052898** | 0.987313281 | 0.772274619 |
| Pseudoperkinsus | **1.58E-07** | 1 | **6.82883E-06** | 8.3954E-06 |
| Thraustochytriidae sp. SEK 706 | **1.72E-07** | 1 | **8.77056E-06** | **1.07677E-05** |
| IN2411 | **1.92E-07** | 1 | **9.32394E-06** | **1.14428E-05** |
| Gastrotricha | **1.16E-06** | 1 | **2.5407E-05** | **3.08888E-05** |
| Coscinodiscus | **1.75E-06** | 0.278986983 | 0.582821031 | 0.57719171 |
| Choreotrichia | **1.51E-05** | 0.311856566 | 0.101541623 | **0.015488844** |
| Actinocyclus | **0.000384573** | 0.250071338 | **0.068692704** | 0.447281642 |
| Prostomatea | **0.00087576** | **0.026671893** | **0.002899562** | 0.267217068 |
| Protaspidae | **0.002038746** | 0.257675163 | 0.416088256 | 0.733365782 |
| Pezizomycotina | **0.004575435** | **0.041127152** | **0.045763065** | 0.952359239 |
| Prasinopapilla | **0.005051704** | **0.040133589** | 0.057107368 | **0.000701025** |
| Oligotrichia | **0.007951565** | 0.585152575 | 0.126665093 | 0.300665478 |
| Rotifera | **0.019756879** | 0.095949987 | 0.329274356 | **0.0157313** |
| Thoracosphaeraceae | **0.022303143** | 0.10577697 | 0.848001144 | 0.145477192 |
| Peridiniales | **0.043640008** | **0.023959761** | 0.445778697 | 0.099130382 |
| Bacillariophyceae | 0.063951412 | **0.037102329** | 0.107855051 | 0.550000607 |
| uncultured Rhizaria | 0.107619637 | 0.28222739 | 0.980032341 | 0.272189874 |
| Oligohymenophorea | 0.141410988 | 1 | **1.55344E-06** | **1.88138E-06** |
| Gymnodinium clade | 0.227526367 | 0.613485081 | 0.310881094 | 0.141918969 |
| Sinophysis | 0.257579735 | 0.766706945 | 0.419052425 | 0.276818712 |
| Gonyaulacales | 0.377379312 | 0.425025336 | **0.043415336** | 0.168797813 |
| Suessiaceae | 0.784372266 | 0.782370869 | 0.826075607 | 0.621305289 |
| Mediophyceae | 0.876815473 | 0.319322954 | 0.986183304 | 0.311589514 |

*Taxa with a p value <0.05 are bold faced.*
